# LGBTQ + Affirmative CBT: a hierarchical linear model of longitudinal outcomes and mechanisms of change

**DOI:** 10.1186/s40359-025-03314-7

**Published:** 2025-09-08

**Authors:** Shelley L. Craig, Vivian W. Y. Leung, Jenny A. Hui, Gio Iacono, Rachael V. Pascoe, Frank Dillon, Ashley Austin, Nelson Pang, Cheryl Dobinson, Ashley S. Brooks

**Affiliations:** 1 Factor-Inwentash Faculty of Social Work, University of Toronto, Toronto, ON M5S 1V4 Canada; 2 School of Social Work, University of Connecticut, 38 Prospect Street, Hartford, CT 06103 USA; 3 School of Social Work, Barry University, 11300 NE 2nd Avenue, Miami Shores, FL 33161 USA; 4 Planned Parenthood Toronto, Toronto, ON Canada; 5 Arizona State University School of Social Work, 411 N. Central Avenue, Suite 800, Phoenix, AZ 3920, 85004 USA; 6 Ontario Institute for Studies in Education, University of Toronto, 252 Bloor Street West, Toronto, ON M5S 1V4 Canada; 7 Taddle Creek Family Health Team, 790 Bay St. Suite 306, Toronto, ON M5G 1N8 Canada

**Keywords:** LGBTQ, CBT, Adolescents, Depression, Longitudinal, Groups, Mechanisms of change, Anxiety, Hope, Minority stress

## Abstract

**Background:**

Sexual and gender diverse adolescents and young adults (SGDAYA) experience mental health disparities, yet few empirical investigations into the long-term impact of affirmative treatments on their well-being exist.

**Methods:**

This study explored the longitudinal effects of a brief affirmative cognitive-behavioral therapy (CBT) group intervention (AFFIRM) on the depression and anxiety of SGDAYA (*N* = 202), as well as how pre-treatment and mid-intervention change mechanisms contributed to their improved mental health. Participants’ age ranged from 14 to 29 years old at baseline (*M* = 22.12, *SD* = 4.60). Data were collected at four time points (pre-test, post-test, 6 months, 1 year) and analyzed using hierarchical linear models.

**Results:**

Participants reported significant improvements in anxiety and depression from baseline to 1-year follow-up as well as increased engagement coping, social support, hope, and improved stress appraisal. SGDAYA, who appraised stress as a threat and had less ability to envision a hopeful future (hope pathway) at baseline, reported greater improvements in depression and anxiety; additionally, those who used more disengagement coping strategies prior to AFFIRM reported more reduction in depression. Participants with the most significant long-term improvement in depression reported (a) greater increases in their resources to deal with stress, (b) more uptake of engagement coping, and (c) improved hope pathway.

**Conclusions:**

This study suggests that an affirmative cognitive-behavioural group intervention designed for SGDAYA can have a long-term impact on their depression and anxiety and highlights the important role of engagement coping, social support, hope and cognitive appraisals on youth mental health.

**Trial registration:**

AFFIRM was retrospectively registered as a clinical trial on March 24th, 2020 (identifier: NCT04318769).

Sexual and gender diverse adolescents and young adults (SGDAYA) experience significant mental health disparities [1]. Compared to their cisgender, heterosexual counterparts, SGDAYA American college students are twice as likely to report significant depression and three times more likely to have suicidal ideation [2]. According to a recent meta-analysis, sexual minority youth have three times the odds of developing a depressive disorder or symptoms compared to their heterosexual peers [3]. Further, SGDAYA experience significantly higher rates of anxiety and depression symptoms compared to heterosexual and cisgender peers. Among all SGDAYA—transgender and nonbinary (TNB) youth, and youth with “emerging identities” such as pansexual and demisexual, report the highest levels of anxiety symptoms relative to their heterosexual and cisgender counterparts [4]. A national study found that 72% of SGDAYA respondents—including over 75% of TNB youth—reported symptoms of generalized anxiety disorder in the past two weeks [5]. Moreover, between 49 and 62% of TNB youth and young adults met the diagnostic criteria for a depressive disorder [6] and over 40% attempted suicide during adolescence or young adulthood [7].

The mental health disparities of SGDAYA are typically attributed to minority stress, described as the unique stressors emerging from a stigmatised sexual orientation [8] or gender identity [9]. Minority stress theory captures the ways in which distal and proximal identity-based stressors exacerbate mental and behavioural health risk through prejudicial events, expectation of rejection, identity concealment and internalized stigma [10]. SGDAYA are more likely to endure distinct minority stressors that exacerbate psychosocial distress—including increased familial rejection, victimisation, and social exclusion compared to their cisgender, heterosexual peers [11–15] as well as higher rates of adverse childhood events [16, 17]. Recent studies have found that sexual minorities frequently seek help for anxiety and depression, yet do not always receive appropriate care [18]. To address the troubling disparities in mental health, affirmative psychotherapies (which acknowledge and support the identities and experiences of sexual and gender diverse clients) [19, 20], have been developed [21], yet there is a lack of empirical research on their implementation. Longitudinal studies and those that parse out the contributions of coping and risk factors to mental health [22] and explore affirmative mechanisms of change [23], remain scarce. This study explored the longitudinal impact and change mechanisms of AFFIRM, an affirmative cognitive behavioral group (CBT) group intervention developed systematically with SGD stakeholders [24] and tested within “real-world” community settings [25, 26]. Through a manualized eight-session curriculum, AFFIRM emphasizes the core principles of CBT while addressing the specific developmental needs of SGDAYA who contend with a daily onslaught of minority stress [25].

## Stress appraisal, engagement coping, & hope

SGDAYA encounter both minority stressors and universal adolescent stressors which can impact mental health through negative cognitive, affective, and behavioural processes [21, 27]. Several factors are emerging as potentially important mechanisms for promoting positive changes in affirmative therapeutic approaches including stress appraisal, engagement coping strategies, and hope development [26]. Appraisal, the process of assessing situations related to personal wellbeing, contributes to individual behaviours and emotions [28]. Such appraisals can manifest as perceiving stress as either a threat or a challenge. Appraising stress as a threat (threat appraisal) includes evaluation of the harmfulness, likelihood, and severity of an event [29] and has been linked to maladaptive coping, high anxiety and poor mental health. Appraising stress as a challenge allows for positive reinterpretation that can contribute to active coping and healthy adjustment, and therapeutically, CBT has been found to improve threat reappraisal [30]. Because SGDAYA experience persistent stressors, it is important to help youth shift their ability to appraise appropriate experiences as challenges that can be managed (i.e., engage in conscious actions to regulate behaviour and thoughts when under stress [31]). Shifts in cognitive appraisals can positively impact the emotional and behavioural health of SGDAYA [32].

Mounting data identifies a range of strategies for coping with stressors and the differential benefits of engagement coping, sometimes referred to as functional coping responses (e.g., active coping, planning, using instrumental support, using emotional support, venting, self-distraction, positive reframing, humor, acceptance, and religion) compared to disengagement or avoidant coping responses (e.g., behavioural disengagement, self-blame, denial, and substance use [33–35]). Reliance on avoidant coping strategies to deal with stressors is linked with depression and suicidality among gender diverse populations [3]. However, in a study based on the stigma coping framework, SGDAYA benefited from engagement coping strategies such as cultivating emotional support (e.g., expressing their feelings to safe others), providing instrumental support (e.g., helping peers deal with a challenging situation), positive framing (e.g., shifting perspective to find growth in challenge), and planning coping (e, g., planning ahead to manage challenges) [25]. The importance of engagement coping dovetails with the established benefits of group interventions for SGDY (mutual aid, peer support, and learning) [35], which can accelerate mental health benefits.

Hope is a future-oriented, cognitive-emotional construct that has been defined as the interaction of pathways thinking (ability to identify goals and the problem-solving strategies needed to achieve them) and agency thinking (belief in one’s ability to initiate and maintain goal directed behaviors [36, 37]. Hope is associated with greater life satisfaction and positive mental health, particularly fewer stress or anxiety disorders, among general population adolescents and young adults [38], as well as among SGDAYA [39]. Those with high levels of hope are more resilient in the face of challenges, perceive stressors as manageable and recover more quickly when challenged [36]. Moreover, an integrative review indicated that hope is associated with improved engagement coping and healthy behaviors and serves as protective factor against suicidality and depression in the face of negative life events [40]. Given the high rates of hopelessness, depression, and suicidality among SGDAYA, hope may represent an important resilience factor.

### Affirmative cognitive behavioural therapy

Affirmative cognitive behavioural therapy (CBT) builds on the efficacy of traditional CBT to treat mental health problems [41] in a model that centers participants stigmatized identities and experiences. Affirmative CBT is based on foundational CBT theories, however given the lack of CBT outcome data related to SGDAYA, the lack of inclusion of minority stress in CBT and studies that demonstrate the impact of interventions tailored to SGDAYA, we previously adapted CBT for SGDAYA [24]. Affirmative CBT actively validates marginalized identities by acknowledging the impact of interpersonal and structural sources of SGD identity-based stigma and targets the cognitive, affective, and behavioural processes related to the internalization and manifestation of this stigma. It is also an evidence-based approach to reducing depression and improving short-term coping among SGDAYA ages 14–25 [25, 27]. Through a range of SGD-specific interventions and skills, affirmative CBT can effectively address complex stressors that exacerbate depression and psychological distress for SGDAYA [3]. Affirmative interventions that mobilize SGDAYA coping skills to help them identify, evaluate, and interrupt the influence of minority stress on their behavioural health are increasingly influential [26], yet longitudinal research is needed.

CBT has demonstrated favourable long-term effects on the mental health of youth and young adults. One meta-analysis focused on longitudinal studies of youth who received CBT for anxiety, depression, or traumatic stress [42]. Across 79 articles, the researchers found that the beneficial effects of CBT lasted from post-treatment, 1-month, 3-month, 6-month, 1-year, through to long-term (2 or more years) follow-up [42]. In an evaluation of the effectiveness of CBT for youth who completed treatment for anxiety disorders an average of 6.17 years earlier, the researchers found that 85.7% of participants no longer met diagnostic criteria for any anxiety disorder, and several benefits shown at 12-month follow-up were maintained years later [43]. Further, individual CBT and group CBT both demonstrate long-term benefits for youth. In another study [44], youth with anxiety disorders received individual or group CBT in a community setting. Two to five years post-treatment, 63% of the youth no longer met criteria for the principal anxiety diagnosis, and most participants reported reductions in anxiety symptoms [44]. Gay men living with HIV who participated in CBT groups reported lower levels of depression and greater perceived social support up to 12 months post-intervention [45]. Moreover, CBT may produce treatment outcomes with greater durability than other interventions. Anxious youth who received CBT treatment during early adolescence reported less severe depressive symptoms two years later, relative to anxious youth who were not treated or who received an alternative, child-centred therapy [46].

Studies of the longitudinal effects of affirmative CBT are far less common, and extant research typically assesses participants a few months after they receive the intervention [47]. A study focused on the delivery of affirmative CBT (i.e., Effective Skills to Empower Effective Men; ESTEEM) to young gay and bisexual men [48] found that compared to waitlist, participants reported lower levels of depression, fewer risky sexual behaviours, and greater reductions in heavy drinking after three months. Results were greatest for those that indicated higher levels of internalized homonegativity at baseline. Lastly, research suggests that SGDAYA may experience long-term benefits from affirmative CBT delivered through virtual platforms. For instance, “Rainbow SPARX” is a virtual self-help program that offers an affirmative CBT approach to SGDAYA experiencing depressive symptoms; participants reported lower levels of depression at three months post-intervention [49].

In addition to a greater understanding of the long-term benefits of affirmative CBT, research elucidating the mechanisms of therapeutic change associated with effective CBT interventions is needed. Within CBT effectiveness studies there has been an interest in exploring change mechanisms for the purpose of strengthening the processes undergirding recovery and symptom improvements [37]. Research has begun to identify common mechanisms of change in CBT interventions with general population samples. For example, anxious youth report alleviated symptoms of anxiety after participating in CBT, achieved through mechanisms such as gradual exposure to a feared stimulus, cognitive restructuring (e.g., changing self-talk), relaxation techniques, and development of a therapeutic alliance [50]. Similarly, CBT affects beneficial changes among depressed youth through cognitive restructuring (e.g., identifying and modifying unhelpful automatic thoughts), strong therapeutic alliances, and therapists’ adherence to and competence with CBT techniques [51]. Cognitive restructuring also underlies therapeutic changes in trauma-focused CBT; specifically, adult participants in the United Kingdom (U.K.) experienced reductions in PTSD symptoms after they identified and changed dysfunctional appraisals (i.e., perceptions that threaten the individual’s view of the self and the world) [52]. Another mechanism for therapeutic change in CBT occurs when the participant acquires and practices skills for coping, including cognitive restructuring, thought records, and pursuing enjoyable activities [53].

Given the emergent focus on research exploring mechanisms for therapeutic change [54, 55] and the importance within SGD populations [56], this study will focus on those processes for SGDAYA engaged in affirmative CBT. In particular, we will explore the potential role of stress appraisal, adaptive coping strategies, and hope as potential mechanisms for positive change.

### Present study

Given the need for supportive interventions for SGDAYA [57] and the dearth of research exploring the long-term efficacy of affirmative CBT interventions [47], this study aimed to: (a) Identify the longitudinal impact of AFFIRM participation on the depression and anxiety of SGDAYA; (b) Specify the pre-treatment variables that contribute to increased longitudinal changes in depression and anxiety; and (c) Explore the relationship between changes in key intervention constructs (hope, stress appraisal, coping) on depression and anxiety to identify the mechanisms of change.

## Method

The current study reports data from the AFFIRM registered clinical trial, with the detailed study design described elsewhere [26, 58]. This study delves into the longitudinal outcomes and change mechanisms of the AFFIRM intervention. Recruitment was conducted (2018–2020) using social media advertisements, through listservs of partnering local organizations and a dedicated project website. SGDAYA were eligible to participate if they: (a) were 14–29 years old at time of enrollment, (b) self-identified as a sexual and/or gender minority, (c) self-identified with depression or mental health stressors, and (d) were proficient in English. Potential participants were excluded from the study if they were assessed to be in crisis (e.g., at high risk of current suicidality) or otherwise warranting a more intensive intervention. Eligible participants were contacted via e-mail by the research coordinator and provided a link to a Qualtrics survey containing informed consent, study details, and pre-test; then, the participant was allocated to the intervention or waitlisted control, as described previously [26]. Participants in a single group varied in age by no more than five years, with cut-offs of 14–18 (5 groups), 19–24 (9 groups), and 25–29 (15 groups) to reflect differences in life stage. The groups utilized the same intervention manual delivered over eight weeks, but examples and exercises were adapted to match the group’s developmental stage (e.g., school scenarios for youth modified to work scenarios for young adults when applying thought stopping). Group size ranged from 4 to 14 participants with a mean of 8.38, median of 8, and mode of 8. Participants completed measures at four time points: (a) T1, pre-test (i.e., immediately prior to beginning an AFFIRM group); (b) T2, post‐test (e.g., immediately following the final group); (c) T3, six months; and (d) T4, one year after completion of AFFIRM. As compensation for completing the questionnaires, participants received e‐gift cards (T1: $25 at pre‐test, T2: $30 at post‐test, T-3: $ 35, and T4- $40). Study procedures were approved by the University of Toronto. Results of the AFFIRM clinical trial showed positive immediate post-therapy outcomes (significant reduction in depression and improvements in intervention factors such as coping, stress appraisal, and hope) compared to control with medium to large effect sizes [26].

### Participants

To elucidate the process of change, this paper highlighted AFFIRM participants who attended the in-person sessions between April 2018 and March 2020. Within the sample, 145 participants (71.8%) attended more than half of the intervention (i.e., five sessions or above). Intent-to-treat analysis was used, so that participants who dropped out (28.2%) were still included in the sample. The most common reasons for attrition were scheduling or transportation challenges. This resulted in a total sample size of 202 from 29 different AFFIRM groups. Participants’ age ranged from 14 to 29 years old at baseline (*M* = 22.12, *SD* = 4.60) with a range of gender, sexual and racial, ethnic identities (detailed in Table 1).

**Table 1:** Demographic characteristics of participants (*n* = 202)

Characteristics	*n*/M	%/SD
Age (at baseline)	22.12	4.60
Gender Identity (most important)^a^		
Transgender	47	23.3
Non-Binary	42	20.8
Cis man	38	18.8
Cis woman	38	18.8
Queer	16	7.9
Agender	10	5.0
Two-Spirit	2	1.0
Other	9	4.5
Sexual Orientation (most important)		
Gay	50	24.8
Queer	46	22.8
Pansexual	31	15.3
Bisexual	30	14.9
Lesbian	22	10.9
Ace Umbrella ^b^	7	3.5
Straight	4	2.0
Not Sure/Questioning	4	2.0
Demi/Demiromantic	3	1.5
Other	5	2.5
Ethnic/Racial Identity ^c^		
White	110	54.5
Asian	47	23.3
Black	39	19.3
Multi-ethnic	30	14.9
Hispanic/Latinx	17	8.4
Indigenous	11	5.4
Middle Eastern	10	5.0

Note. ^a^ Most important represents their most important gender or sexual identity at the time of data collection ^b^Ace umbrella represents spectrum of asexuality. ^c^ Categories were not mutually exclusive

### Intervention

The AFFIRM intervention includes: an orientation (group norms, confidentiality, curriculum); sessions 1–2 (overview of CBT and the impact of minority stressors); sessions 3–4 (CBT, thought stopping, stress appraisal); sessions 5–6 (fostering adaptive coping skills, goal setting and cultivating feelings of hope); and sessions 7–8 (social support, self-compassion, self‐care plans). AFFIRM provides opportunities for SGDAYA to be more aware of, address, and change cognition (self-awareness, identifying stress, changing thoughts about managing stress), mood (recognising the link between thoughts and feelings, identifying future oriented thoughts and goals that promote positive feelings and hope for the future), and behaviour (identifying strengths, assessing current coping strategies, integrating more adaptive coping strategies). Through AFFIRM, SGDAYA learn to better understand sources of stress—including minority stress (e.g., media messages, family rejection, school bullying)—and engage in a variety of cognitive and behavioural strategies to unlearn, challenge, and question stigmatising messages and beliefs. Participants engage in a range of experiential group CBT activities hypothesized to: (a) validate the authenticity of their challenging experiences; (b) improve youths’ ability to locate the problem outside of themselves (e.g., there is something wrong with homophobia/transphobia) rather than within themselves (e.g., there is something wrong with me); (c) build participants’ capacity to recognize stress as a normal, albeit challenging, part of life that can be managed with effective coping strategies rather than as a threat over which they have no power or control; (d) foster the development of healthy and adaptive coping strategies particularly relevant to the lives of SGDAYA (e.g. building identity-affirming social support systems, goal setting and behavioural activation, and engaging hope).

The AFFIRM groups were co-facilitated by two graduate level clinicians with a minimum of two years of training that completed the 16-hour AFFIRM facilitator training [59] and had delivered AFFIRM at least once prior to the study. AFFIRM facilitators had a range of intersectional identities (e.g., transgender, lesbian, bisexual, cisgender, gay, gender diverse) and all had significant clinical group experience. A clinical supervisor also provided coaching for the study duration.

### Measures

#### Stress appraisal

The 13-item Stress Appraisal Measure for Adolescents (SAMA) was used to measure participants’ daily stress appraisal as challenge, threat, and resources [60]. The scale was rated by a 5-point Likert scale (1 = strongly disagree to 5 = strongly agree) with higher scores indicating greater endorsement. A sample item is “Stressful events have serious implications for my life”. The challenge subscale measured participants’ likelihood to appraise stress as challenge (3 items, α = 0.85); the threat subscale measured their tendency to appraise stress as threat (7 items, α = 0.84), and the resources subscale indicated the resources they possessed to deal with stress (3 items, α = 0.83). The three subscales were totalled separately and mean centered.

#### Coping

The 28-item Brief COPE Inventory (BCI) was used to measure participants’ coping strategies [61]. Responses ranged from 1 (not at all) to 4 (a lot), and a sample item is “I look for something good in what is happening”. The original BCI contained 14 coping subscales, but we followed the three-factor structure [62] for ease of interpretation. The three factors included: (a) engagement coping (12 items, α = 0.74), consisted of positive reframing, planning, acceptance, active coping, self-distraction, and humor; (b) disengagement coping (6 items, α = 0.71), including denial, behavioural disengagement, and self-blame; and (c) social support coping (8 items, α = 0.77), containing emotional support, instrumental support, venting, and religion. The three subscales were totalled separately and mean centered.

#### Hope

The 12-item Hope Scale (HS) was administered to represent participants’ level of hope [63], ranging from 1 (definitely false) to 8 (definitely true). A sample item is “There are lots of ways around any problem”, and a higher score represents a higher level of hope. The scale consists of two subscales with four items each: the agency subscale measures participants’ goal-directed determination (α = 0.84), and the pathway subscale measures how likely the respondents plan ways to meet their goals (α = 0.82). The two subscales were totalled separately and mean centered.

#### Depression

The level of depression was measured by the Beck Depression Inventory-II (BDI-II) [64]. The BDI-II is a 21-item instrument based on participants’ self-report of their experiences in the previous two weeks. Items were scored from 0 to 3, and a sample item included “I am dissatisfied or bored with everything”. The total scores (ranging between 0 and 63) were calculated for analyses, and a higher score indicated higher levels of depression. In the present study, the scale had excellent reliability (α = 0.93) and all scores were mean centered.

#### Anxiety

Three corresponding questions for anxiety from the DSM-5 Self-Rated Level 1 Cross-Cutting Symptom Measure – Child [65] were totaled to measure participants’ level of anxiety. A sample question was: “During the past two weeks, how much (or how often) have you felt nervous, anxious, or scared?”, and responses were scored from 1 (not at all) to 5 (nearly every day). The scale demonstrated good internal consistency in this study (α = 0.83) and all scores were mean centered.

### Demographic characteristics

Age, gender identities (most important), sexual orientation (most important), and ethnic/racial identities (non-mutually exclusive) were collected. As described in the trial protocol [58], as an approach to capture intersectional identities, our demographic data collection involved two steps: (a) endorsing all identities that represented their sexual and gender identities and (b) endorsing one identity that was most important to them at the time. This approach allows for some comparison between groups by encouraging the participants (instead of the researchers) to identify their most important identity while simultaneously acknowledging intersectionality.

### Data analysis plan

The missing data ranged from 0 to 2.7% at the item level. The Expectation-Maximization (EM) Algorithm in SPSS 28 was used to replace the missing items of BDI-II. Mean scores were calculated for other scales, so average scores were obtained from all remaining items unless participants missed the entire scale. Multicollinearity testing (Variance Inflation Factor [VIF] and Tolerance statistics) was performed using linear regression models that included all predictors used in the log-transformed multilevel models. Results indicated that multicollinearity was generally within acceptable bounds, with most predictors showing VIFs well below the conventional threshold of 5. The only exception was hope (agency), which exceeded this threshold (VIF = 5.32). This coefficient should therefore be interpreted with caution.

### Overall changes between baseline to one-year follow-up

To examine longitudinal (a) changes of AFFIRM participants’ outcomes and (b) changes of AFFIRM intervention mechanism variables across four time points, hierarchical linear models with restricted maximum likelihood estimation (REML) were created to test the effects of time, with age as a covariate. Participants’ changes were hypothesized to follow a log curve (i.e., steep change between baseline and post-test due to the intervention and stable in six-month and one-year follow-up) based on prior theoretical and empirical work. CBT often produces early intraindividual gains in psychological mechanisms such as hope, with the steepest change occurring within the first half of treatment [37]. AFFIRM similarly targets core transdiagnostic mechanisms in a brief format and thus would be expected to produce the largest gains during the intervention period. Although CBT can also produce sustained improvements post-intervention in general clinical populations [42], AFFIRM’s SGDAYA participants experience chronic proximal and distal minority stress, which has been shown to attenuate post-intervention improvement as participants re-engage with and ruminate upon environments that produce minority stress [23, 66, 67]. Because AFFIRM includes no booster or follow-up programming, and because log-transformed time models capture decelerating change while maintaining parsimony, a log-linear model was selected as the most theoretically appropriate functional form. This approach also avoids the overfitting risks posed by more flexible but less constrained models such as piecewise growth modeling [68].

The time points were scored as: baseline = log_e_ (1) = 0, post-test = log_e_ (2), six-month follow-up = log_e_ (3), and one-year follow-up = log_e_ (4). Time point entries were nested under participant ID, and participants were further nested according to their AFFIRM group to account for possible interdependence of data based on group. Intraclass Correlation Coefficients (ICC) were estimated to understand the proportion of total variance attributed to within-subject variance. Age was included as a covariate variable in the model, centred at the mean of the sample. Effect sizes were calculated using Feingold’s d, which standardizes unstandardized fixed effect estimates by dividing them by the product of the baseline standard deviation and the square root of twice one minus the estimated test-retest correlation (approximated by the ICC):

$$\:d=\frac{{\beta\:}}{SD\cdot\:\sqrt{2\left(1-r\right)}}$$

This method accounts for the repeated measures design and provides a standardized estimate of the within-subject effect size for each time contrast. Due to multiple testing, the false discovery rate (FDR) was used to adjust the significance of coefficients [69, 70].

### Roles of AFFIRM intervention variables in mental health impacts of AFFIRM

Stress appraisal, coping, and hope were considered as AFFIRM intervention variables, and depression and anxiety were analyzed as outcome variables in multilevel linear models. Three models (one for each group of AFFIRM intervention variables) were created for each outcome variable, resulting in six models in total. Similar to the first step, all models included age (centred at sample mean) as a covariate, and participants were clustered based on the AFFIRM groups.

First, baseline scores of AFFIRM intervention variables and their interaction terms with time (e.g., Time × stress appraisal at baseline) were included in the models to estimate how the slopes (rates of change of outcome variables) were affected by the baseline scores of AFFIRM intervention variables. Second, post-test scores of AFFIRM intervention variables and their interaction terms with time (e.g., Time × engagement coping at post-test) were also included to examine how changes in AFFIRM intervention variables between baseline and post-test relate with the rate of changes of outcome variables. By having both baseline and post-test scores and their interaction terms in the same model, the post-test scores (after controlling for baseline) signify the changes of AFFIRM intervention variables across the two time points. This thus answered the second question: Did the changes of AFFIRM intervention variables between baseline and post-test have long-term effects on outcome variables after one year?

## Results

### Overall changes between baseline to one-year follow-up

Table 2 shows the descriptive statistics of all variables across four time points and the ICCs. After controlling for age and adjusting for multiple testing, participants reported significant improvements in all outcomes from baseline to post test with results maintained to 1-year follow-up. Specifically, they reported increased use of engagement coping (*B* = 0.10, *p* < .001) and increased social support (*B* = 0.17, *p* < .001) and were more likely to appraise stress as challenge (*B* = 0.48, *p* < .001) and had the resources to deal with stress (*B* = 0.40, *p* < .001). As well, there were increases in hope, both agency (*B* = 0.24, *p* = .010) and pathway (*B* = 0.26, *p* = .003). Also, at one year after the intervention, participants showed reductions in depression (*B* = -2.36, *p* < .001), anxiety (*B* = -0.28, *p* < .001), and disengagement coping (*B* = -0.11, *p* = .002), and they were less likely to appraise stress as threat (*B* = -0.23, *p* < .001). The effect sizes ranged from 0.26 to 0.79, indicating approximately small to medium effect sizes.

**Table 2:** Descriptive statistics and overall time effects (*n* = 202)

	ICC			Baseline (*n* = 196)	Post-test (*n* = 160)	6-month follow-up (*n* = 90)	1-year follow-up (*n* = 91)	Time effect (log)	
	WP	BP	BG	*M (SD)*	*M (SD)*	*M (SD)*	*M (SD)*	Est.	*d*
Depression	0.67	0.67	0.25	21.06 (11.88)	17.14 (12.56)	19.26 (12.85)	18.55 (13.17)	-2.45^***^	-0.25
Anxiety	0.48	0.52	0.17	3.55 (1.07)	3.20 (1.11)	3.34 (1.06)	3.17 (1.03)	-0.27^***^	-0.25
Stress Appraisal									
Challenge	0.45	0.50	0.12	3.03 (0.97)	3.68 (0.94)	3.58 (1.03)	3.58 (1.01)	0.48^***^	0.47
Threat	0.47	0.51	0.14	4.10 (0.66)	3.81 (0.73)	3.91 (0.61)	3.86 (0.80)	-0.23^***^	-0.34
Resources	0.57	0.56	0.12	3.65 (0.91)	3.98 (0.92)	3.96 (0.91)	3.97 (0.93)	0.39^***^	0.46
Coping									
Engagement	0.57	0.59	0.82	2.78 (0.44)	2.95 (0.46)	2.94 (0.46)	2.91 (0.43)	0.12^***^	0.29
Disengagement	0.59	0.57	0.13	2.51 (0.58)	2.36 (0.57)	2.41 (0.62)	2.35 (0.61)	-0.11^**^	-0.21
Social Support	0.58	0.58	0.80	2.46 (0.57)	2.67 (0.61)	2.65 (0.61)	2.64 (0.54)	0.20^***^	0.38
Hope									
Agency	0.62	0.64	0.18	4.52 (1.64)	5.07 (1.65)	4.81 (1.60)	4.66 (1.70)	0.31^**^	0.22
Pathway	0.55	0.59	0.13	5.13 (1.45)	5.68 (1.39)	5.57 (1.41)	5.36 (1.35)	0.28^**^	0.20

Note. *p < .05; **p < .01; ***p < .001. WP = within-person ICC; BP = between-person ICC; BG = between-group ICC. After FDR adjustment, time effects were significant for all measures

### Pre-treatment variables and mental health

The interaction terms of time × baseline scores of intermediate variables indicated the relationship between pre-test scores and overall AFFIRM benefits. According to Table 3, participants reported more reduction in depression and anxiety over one year if they appraised stress as threat (Depression: *B* = -3.43, *p* = .037, *d* = -0.35; Anxiety: *B* = -0.48, *p* = .01, *d* = -0.44) and had lower scores in pathway (Depression: *B* = 2.06, *p* = .018, *d* = 0.18; Anxiety: *B* = 0.30, *p* = .003, *d* = 0.30) at baseline. Participants who reported higher scores in disengagement coping at baseline also reported more reduction in depression (*B* = -5.16, *p* = .003, *d* = -0.47), but not anxiety (*B* = -0.35, *p* = .076, *n.s.*). Based on the *d* values, the effect sizes were approximately medium.

#### Process of change: post-test scores as covariates

The interaction terms between time and post-test scores of intermediate variables indicated the relationship between the changes of intermediate variables (between baseline and post-test) and the changes of outcome variables (from baseline to one-year follow-up). For depression, the significant interaction terms were time with resources (*B* = -3.42, *p* = .007, *d* = -0.35), engagement coping (*B* = -7.95, *p* = .004, *d* = -0.73), and pathway (*B* = -1.98, *p* = .037, *d* = -0.18). The results suggest that participants reported greater improvements in depression one year after AFFIRM if they showed the following changes between baseline and immediately after AFFIRM: (a) greater increases in their perceptions of the resources they have to deal with stress, (b) more uptake of engagement coping, and (c) greater increases in hope pathways to attain their goals. For anxiety, none of the interaction terms were significant, meaning that the longitudinal changes in anxiety were not related to the changes of the intermediate variables. The Feinberg’s *d*s indicate small to medium effect sizes for the relationships found above.

**Table 3:** Results of process of change models and model fit statistics (*n* = 202)

	Depression						Anxiety					
Model 1 & 2: Stress Appraisal	Est.	95% CI				d	Est.	95% CI				d
Age	-0.34*	-0.66, -0.02				-	-0.01	-0.04, 0.02				-
Time	9.88	-8.17, 27.92				1.02	0.75	-1.3, 2.79				0.69
Challenge (baseline)	-2.70*	-4.95, -0.45				-	-0.21	-0.43, 0.01				-
Threat (baseline)	3.08	-0.09, 6.25				-	0.47**	0.16, 0.78				-
Resources (baseline)	-4.14**	-6.73, -1.55				-	-0.15	-0.41, 0.1				-
Time * Challenge (baseline)	1.03	-1.12, 3.18				0.11	0.11	-0.13, 0.36				0.10
Time * Threat (baseline)	-3.43*	-6.64, -0.22				-0.35	-0.48**	-0.85, -0.12				-0.44
Time * Resources (baseline)	2.48*	0.09, 4.88				0.26	0.14	-0.13, 0.42				0.13
Challenge (post-test)	-1.60	-4.15, 0.96				-	-0.03	-0.28, 0.22				-
Threat (post-test)	2.33	-0.38, 5.03				-	0.29*	0.02, 0.56				-
Resources (post-test)	0.36	-2.35, 3.06				-	0.01	-0.25, 0.27				-
Time * Challenge (post-test)	-0.86	-3.27, 1.54				-0.08	-0.01	-0.28, 0.26				-0.01
Time * Threat (post-test)	1.73	-1.07, 4.52				0.17	0.20	-0.12, 0.53				0.23
Time * Resources (post-test)	-3.42**	-5.91, -0.94				-0.35	-0.15	-0.44, 0.13				-0.16
	**AIC**				**BIC**		**AIC**				**BIC**	
	2906.19				2922.09		1109.47				1125.36	
	**R²**_m_				**R²**_C_		**R²**_m_				**R²**_C_	
	0.39				0.75		0.23				0.59	
**Model 3 & 4: Coping**	**Est.**	**95% CI**				***d***	**Est.**	**95% CI**				***d***
Age	-0.07	-0.39, 0.25				-	0.00	-0.03, 0.03				-
Time	10.56	-5.69, 26.82				1.09	-0.44	-2.27, 1.40				-0.40
Engagement (baseline)	-1.88	-7.04, 3.29				-	0.19	-0.33, 0.72				-
Disengagement (baseline)	8.81***	5.46, 12.16				-	0.57**	0.22, 0.91				-
Social Support (baseline)	-3.00	-6.70, 0.71				-	-0.20	-0.58, 0.18				-
Time * Engagement (baseline)	5.50*	0.30, 10.7				0.50	0.62*	0.04, 1.21				0.63
Time * Disengagement (baseline)	-5.16**	-8.57, -1.76				-0.47	-0.35	-0.74, 0.04				-0.36
Time * Social Support (baseline)	2.98	-0.55, 6.51				0.27	-0.01	-0.41, 0.39				-0.01
Engagement (post-test)	1.24	-4.16, 6.63				-	-0.04	-0.59, 0.50				-
Disengagement (post-test)	5.47**	1.90, 9.04				-	0.56**	0.19, 0.93				-
Social Support (post-test)	-1.10	-5.1, 2.9				-	0.10	-0.31, 0.51				-
Time * Engagement (post-test)	-7.95**	-13.34, -2.55				-0.73	-0.55	-1.15, 0.06				-0.56
Time * Disengagement (post-test)	2.98	-0.58, 6.54				0.27	0.23	-0.18, 0.63				0.23
Time * Social Support (post-test)	-2.45	-6.35, 1.45				-0.22	0.15	-0.30, 0.59				0.15
	**AIC**		**BIC**				**AIC**		**BIC**			
	2818.48		2834.30				1070.96		1086.78			
	**R²**_m_		**R²**_C_				**R²**_m_		**R²**_C_			
	0.44		0.76				0.26		0.59			
**Model 5 & 6: Hope**	**Est.**	**95% CI**				***d***	**Est.**	**95% CI**				***d***
Age	-0.21	-0.57, 0.15				-	-0.01	-0.04, 0.02				-
Time	-2.73	-9.51, 4.06				-0.28	-0.88*	-1.65, -0.12				-0.81
Agency (baseline)	-1.47	-3.28, 0.34				-	-0.04	-0.22, 0.14				-
Pathway (baseline)	-2.05*	-4.01, -0.10				-	-0.22*	-0.41, -0.03				-
Time * Agency (baseline)	1.33	-0.26, 2.92				0.13	0.02	-0.16, 0.20				0.02
Time * Pathway (baseline)	2.06*	0.36, 3.76				0.18	0.30**	0.11, 0.49				0.30
Agency (post-test)	-2.49*	-4.67, -0.31				-	-0.09	-0.31, 0.12				-
Pathway (post-test)	2.09	-0.22, 4.39				-	0.14	-0.09, 0.37				-
Time * Agency (post-test)	-1.09	-2.86, 0.69				-0.11	-0.12	-0.32, 0.08				-0.13
Time * Pathway (post-test)	-1.98*	-3.84, -0.12				-0.18	-0.08	-0.29, 0.14				-0.08
	**AIC**			**BIC**			**AIC**			**BIC**		
	2899.12			2914.99			1117.42			1133.27		
	**R²**_m_			**R²**_C_			**R²**_m_			**R²**_C_		
	0.25			0.75			0.10			0.60		

Note. *p < .05; **p < .01; ***p < .001. AIC = Akaike Information Criterion; BIC = Bayesian Information Criterion; R²_m_ = marginal R²; R²_C_ = conditional R²

## Discussion

This study identifies the contribution of AFFIRM, the first intervention for SGDAYA that demonstrates long-term effectiveness in reducing depression and anxiety. Given the mental health disparities that affect this population [1], and research demonstrating that 12-month outcomes are predictive of sustained positive mental health [42], the results of this study provide important insight into several critical factors for the delivery of affirmative CBT to SGDAYA. First, the positive effects of AFFIRM were maintained long-term. Overall, participants reported significant decreases in depressive and anxiety symptoms that persisted for one year, as well as significant improvements in key factors of hope, social support, stress appraisal and coping. These findings build on the results of previous studies on the short-term impact of affirmative CBT [25, 26] and suggest that AFFIRM is an effective intervention to support the long-term behavioural health of SGDAYA. These encouraging findings align with studies that identified long term benefits of CBT on youth anxiety and depression post-treatment [44, 45].

Second, this study identifies participants’ baseline characteristics that affect their response to AFFIRM, leading to long-term improvements. SGDAYA who faced multiple challenges prior to AFFIRM (e.g., appraising stress as a threat and perceiving fewer pathways for a hopeful future), reported greater long-term improvements in depression and anxiety after 12 months. SGDAYA who reported higher scores in disengagement coping at baseline also reported more reduction in depression. Similar to other research [71], those with fewer engagement coping strategies at pre-treatment showed the most gains from the AFFIRM intervention. It may be that “broadening one’s overall repertoire of [coping] skills [through CBT]” [35, p. 1555] can be especially crucial for individuals experiencing greater psychosocial distress, as these individuals may require a broader range of skills to deal with challenges that arise during treatment (e.g., negative affect; lapses). AFFIRM participants reported the strengthening of pathways thinking and belief in a hopeful future, which predicted both anxiety and depression. Similarly, research with general population youth found a relationship between the goal-setting components of CBT and youth mental health [72]. Importantly, the appraisal of stress as a threat impacted SGDAYA anxiety and depression. CBT has been found to reduce anxiety through the development of more *adaptive* threat appraisals [73], and AFFIRM’s focus on cognitive restructuring makes an impact on those factors.

Third, this study elucidates the most significant predictors of decreased depression among SGDAYA that completed AFFIRM. Participants tended to report greater improvement in depression after one year if they: (a) experienced more uptake of engagement coping; (b) reported larger increases in their resources to deal with stress; and (c) developed more pathway hope to attain their goals. Although none of these factors were significantly associated with the long-term reduction in anxiety for SGDAYA, the overall transdiagnostic benefits remain. Given that AFFIRM was originally conceptualized as a response to the high rates of depression seen in research and practice with these populations, the decision was made to include more robust measures of depression than other potential comorbidities. Given that anxiety and depression are distinct yet often overlapping mental health profiles and concerns for youth [74], there may be particular mechanisms of change that can be influenced through future research on affirmative interventions that have a stronger focus on anxiety-relevant components.

After participating in AFFIRM, SGDAYA were more likely to use engagement coping, which predicted positive short- and long-term outcomes in depression and anxiety and may be a critical element of affirmative CBT for this population. Consistent with recommendations to improve SGDY mental health [22], AFFIRM was developed to integrate select CBT strategies (e.g. behavioral activation, cognitive restructuring) to enhance engagement coping. AFFIRM’s outcomes support previous findings that demonstrate the critical benefits of engagement coping. For example, a study of anxious youth showed that those who use greater engagement coping formed stronger alliances with therapists, sought solutions to problems, welcomed guidance from others, and were more willing and motivated to engage with CBT procedures—and, in turn, these factors facilitated positive treatment outcomes [71].

AFFIRM taught participants specific coping strategies that had been identified as beneficial in prior research. For example, racial minority men who have sex with men (MSM) and Latinx SGDAYA used engagement strategies (e.g., educating others, setting boundaries, directly confronting sources of stigma) to cope with racism, homophobia, and other forms of discrimination [75]. When confronted with minority stressors, AFFIRM participants learn to utilize multiple engagement coping strategies, such as cognitive self-talk (e.g., “It will get better”); spending time with others in the SGD community; using online media to connect with others or gain new information; encouraging others to use different gender language (e.g., pronouns); beginning one’s gender transition; and watching SGD films and TV or online shows. Helping participants to identify specific engagement coping strategies tailored to their identities seems to be an important element of affirmative CBT.

The findings underscore the likely importance of targeting participants’ appraisals of minority stressors as a potentially important process for promoting mental health among SGDAYA. As cognitive appraisals of stressors influence stress responses, which impact well-being [76], our study finding offers important insights into intervention processes that may particularly impact youth mental health outcomes. There are several cognitive behavioural strategies embedded in AFFIRM that take into consideration structural sources of stress and the subsequent internalizations of those messages (e.g., identifying the source of the negative cognition, “talking back” to negative thoughts) as well as strategies that help SGDAYA feel empowered to combat those unhelpful thoughts (e.g., behavioural activation plans, self-compassion statements) and facilitate the shift from perceiving stress as an overwhelming threat to a challenge that can be addressed. Further AFFIRM’s focus on fostering engagement coping strategies helps participants build the internal coping resources, as well as external sources of support to manage stressors rather than be overwhelmed by them.

Consistent with recommendations for affirmative interventions [39], AFFIRM utilizes a multi-faceted approach to promoting positive mental health by integrating strategies that both reduce feelings of hopelessness and bolster hopefulness for the future. In a study of adults (women over 18) with a range of anxiety disorders, it was found that compared to waitlist, changes in hope were significantly higher among clients receiving CBT, that greater increases in hope led to decreases in clinician and self-rated anxiety, that CBT predicted increases in hope and these changes were seen early in treatment and that changes in hope mediated the effects of treatment on anxiety [37]. Given this validation of its role as a key mechanism of change in CBT, the results of this study support the importance of hope, particularly pathways thinking, in affirmative CBT. AFFIRM develops hope using a variety of clinical activities (e.g., hope boxes, goal-setting) which contribute to improved coping and overall mental health. A systematic review explored the role of hopefulness in youth (aged 14–25) with depression [77]. Although not all of the studies utilized CBT, they found that hope can be enhanced through treatment and is best supported through a strong therapeutic alliance that encourages youth to identify and pursue goals important to them, specifically through talking and activity therapies [77]. Cultivating hope during CBT can help facilitate effective coping and promote recovery [78]. Consistent with this evidence, AFFIRM was developed with the intent to promote hope by engaging in affirming behaviors and positive cognitions.

### Limitations

Several limitations must be noted. Although the measure of anxiety was chosen because of its utility and efficiency in clinical practice, future studies may consider more comprehensive scales. Other data collection methods, in addition to self-report (e.g., behavior or collateral reporting) may be beneficial. The measure of ethnic and racial identities is non-mutually exclusive, and therefore, identities may be captured in multiple places. Intervention outcomes were only identified pre- and post, and thus, temporal measures of the amount or timing of the mechanisms of change (e.g., hope) are not available by session. Further, as some of the six-month and one-year follow-up surveys were completed in 2020 and 2021, the COVID-19 pandemic may have influenced SGDAYA’s psychological well-being, confounding the longitudinal results. However, a qualitative study found that AFFIRM, shifted to online delivery to respond to SGDY needs during the COVID-19 lockdown, provided multiple participant benefits and may have mitigated further declines in functioning [79, 80].

Multiple candidate models of change were examined—including linear, quadratic, piecewise, and log-transformed time—for each outcome domain. While we ultimately present the log-transformed model in the manuscript, this choice was informed by model comparison and theoretical considerations rather than default preference. Although piecewise models demonstrated better statistical fit in some cases (as would be expected given their additional flexibility), they were less aligned with our theoretical expectation of rapid intervention-driven change followed by tapering effects due to persistent exposure to minority stress. Quadratic models were also tested but failed to converge in several models, raising concerns about overfitting and interpretability. Further, alternative modelling strategies could be considered, such as residualized change scores. Given the multicollinearity observed in the agency hope measure, corresponding findings should be interpreted cautiously. Additionally, some nonsignificant interaction effects should not be interpreted as definitive evidence of no moderation. Given sample size constraints, the study may have been underpowered to detect modest interaction effects, and interpretation should consider confidence intervals alongside *p*-values. Future research with larger samples and more evenly spaced follow-ups could examine which model best captures the trajectory of change following LGBTQ-affirmative interventions.

Finally, even with the limited age range of SGDAYA participating in each of the developmentally appropriate groups and the provision of intensive training and coaching to the clinical co-facilitators, there may be additional mechanisms of change such as therapeutic alliance and group factors that partially accounted for participant outcomes. Future research should consider further exploration of such change mechanisms and participant developmental stage as potential moderators of observed intervention effects as well as a deeper understanding of the particular strategies SGDAYA utilize to support their mental health (e.g., affirmative CBT activities, apps, social support) post-intervention.

## Conclusions

This study responds to the call for tailored, longitudinal, and empirically based interventions to address the striking mental health disparities of SGDAYA. As an affirmative CBT intervention, AFFIRM had significant longitudinal impacts on SGDAYA depression and anxiety. This study adds to the intervention literature by exploring the relationship between key mechanisms of change (i.e., stress appraisal, coping, hope) that are important to positive long-term mental health. The findings underscore the utility of CBT-based approaches that (1) affirm the multidimensional spectrum of SGD experiences and identities, (2) attend to minority stressors, (3) actively target maladaptive responses to stressors, and (4) build engagement coping skills. The results of this study underscore the need for targeted public health interventions that address stigma and discrimination and improve access to mental health care for SGDAYA populations.

## Data Availability

The datasets generated and/or analysed during the current study are not publicly available due to participant confidentiality and vulnerability but may be available from the corresponding author who will consult with the REB for reasonable requests.

## Abbreviations

CBT: Cognitive Behavioural Therapy
SGDAYA: Sexual and Gender Diverse Adolescents and Young Adults
